# Construction of a *Geobacter* Strain With Exceptional Growth on Cathodes

**DOI:** 10.3389/fmicb.2018.01512

**Published:** 2018-07-13

**Authors:** Toshiyuki Ueki, Kelly P. Nevin, Trevor L. Woodard, Muktak A. Aklujkar, Dawn E. Holmes, Derek R. Lovley

**Affiliations:** ^1^Morrill Science Center IV, Department of Microbiology, University of Massachusetts, Amherst, MA, United States; ^2^Department of Physical and Biological Sciences, Western New England University, Springfield, MA, United States

**Keywords:** anaerobic respiration, electromicrobiology, bioelectrochemical, citrate lyase, electrosynthesis

## Abstract

Insoluble extracellular electron donors are important sources of energy for anaerobic respiration in biogeochemical cycling and in diverse practical applications. The previous lack of a genetically tractable model microorganism that could be grown to high densities under anaerobic conditions in pure culture with an insoluble extracellular electron donor has stymied efforts to better understand this form of respiration. We report here on the design of a strain of *Geobacter sulfurreducens*, designated strain ACL, which grows as thick (ca. 35 μm) confluent biofilms on graphite cathodes poised at -500 mV (*versus* Ag/AgCl) with fumarate as the electron acceptor. Sustained maximum current consumption rates were >0.8 A/m^2^, which is >10-fold higher than the current consumption of the wild-type strain. The improved function on the cathode was achieved by introducing genes for an ATP-dependent citrate lyase, completing the complement of enzymes needed for a reverse TCA cycle for the synthesis of biosynthetic precursors from carbon dioxide. Strain ACL provides an important model organism for elucidating the mechanisms for effective anaerobic growth with an insoluble extracellular electron donor and may offer unique possibilities as a chassis for the introduction of synthetic metabolic pathways for the production of commodities with electrons derived from electrodes.

## Introduction

The ability of some microorganisms to accept electrons from insoluble extracellular sources to support anaerobic respiration is of biogeochemical significance and can be harnessed for diverse practical applications. However, elucidating the mechanisms for this type of electron transport has been limited by a lack of genetically tractable anaerobes that can be effectively grown in pure cultures with insoluble electron donors.

Examples of biogeochemically significant anaerobic oxidation of insoluble extracellular electron donors include oxidation of reduced humic substances (Lovley et al., 1999; Coates et al., 2002) and direct interspecies electron transfer (DIET) in which methanogens consume electrons either through direct biological electrical connections (Rotaru et al., 2014a,b; Holmes et al., 2017) or conductive materials (Kato et al., 2012; Liu et al., 2012; Chen et al., 2014a,b).

DIET may be of practical significance during anaerobic digestion of organic wastes (Morita et al., 2011; Rotaru et al., 2014b; Shrestha et al., 2014) and many studies have demonstrated how promoting DIET can accelerate and stabilize the anaerobic digestion process (Cheng and Call, 2016; Lovley, 2017b,c). Direct electron transfer from zero valent iron to microorganisms may be an important mechanism for anaerobic corrosion (Dinh et al., 2004; Uchiyama et al., 2010; Enning et al., 2012; Kato et al., 2014). Feeding microorganisms electrons with an electrode is becoming an increasingly attractive possibility for the production of biofuels and other organic commodities, as well as for bioremediation of organic and metal contamination (Thrash and Coates, 2008; Lovley, 2011; Lovley and Nevin, 2013; Rosenbaum and Henrich, 2014; Tremblay and Zhang, 2015; May et al., 2016; Tremblay et al., 2017).

Some strategies for anaerobic growth with extracellular electron donors are not conducive for detailed molecular investigation of metabolism. For example, growth with humic substances or their analogs yields low maximum cell densities of ≤2 × 10^7^ cells per ml (Lovley et al., 1999; Coates et al., 2002). More biomass can be obtained in co-cultures in which the genetically tractable *Geobacter sulfurreducens* grows by oxidizing the reduced soluble humics analog anthrahydroquinone-2,6-disulfonate (Smith et al., 2015) or via DIET (Summers et al., 2010; Shrestha et al., 2013a,b). However, the presence of the electron-donating partner in these co-cultures complicates mechanistic studies. Substantial iron mineral crusts are associated with cells growing by accepting electrons from zero valent iron (Dinh et al., 2004; Uchiyama et al., 2010; Enning et al., 2012).

Pure culture studies on electron transfer to electrodes have been a powerful approach to elucidate mechanisms for electron transfer to extracellular electron acceptors because they provide an inert surface with a well-defined redox potential (Lovley, 2012, 2017a; Shi et al., 2016). For example, *Geobacter sulfurreducens* generates high current densities (Nevin et al., 2008; Yi et al., 2009) and forms thick (ca. 100 μm) biofilms on graphite electrodes serving as an electron acceptor, which has facilitated diverse gene expression, proteomic, and genetic investigations (Holmes et al., 2006; Reguera et al., 2006; Nevin et al., 2009; Franks et al., 2010, 2012; Inoue et al., 2010; Malvankar et al., 2011, 2012a,b; Leang et al., 2013; Chan et al., 2017).

*G. sulfurreducens* is also capable of growing with electrons derived from graphite electrodes (i.e., cathodes). However, cathode biofilms of wild-type *G. sulfurreducens* consume 20-fold less current than they produce on anodes and biofilms do not extend beyond a mono-layer on the cathode surface (Gregory et al., 2004; Strycharz et al., 2011). Much higher current consumption by *G. sulfurreducens* on stainless steel cathodes was reported (Dumas et al., 2008), but we have been unable to replicate those results. Furthermore, even in those studies, cathode biofilms were thin and patchy. Other genetically tractable microorganisms that can also consume electrons from cathodes include *G. metallireducens* (Gregory et al., 2004), *Shewanella oneidensis* (Ross et al., 2011), *Clostridium ljungdahlii* (Nevin et al., 2011), and *Rhodopseudomonas palustris* (Bose et al., 2014). However, all of these microorganisms grow poorly on cathodes, if at all. The cathode biofilms are sparse and current consumption rates are low.

Here, we report on a strain of *G. sulfurreducens* constructed to enable autotrophic growth. Surprisingly, this strain grew much more effectively on cathodes than the wild-type strain, providing a robust, genetically tractable microbe for the study of cathode-based growth.

## Materials and Methods

### Strains and Growth Conditions

*G. sulfurreducens* strain DL-1 was obtained from our laboratory culture collection and was routinely cultured under anaerobic conditions with acetate as the electron donor and fumarate as the electron acceptor, as previously described (Coppi et al., 2001). In order to construct *G. sulfurreducens* strain ACL, the genes *aclA* and *aclB*, which encode the two subunits for the ATP-citrate lyase of *Chlorobium limicola* (Kanao et al., 2001), were synthesized with codon optimization for *G. sulfurreducens* with GenScript (Supplementary Figure S1). A BamHI-EcoRI fragment of the *aclA* and *aclB* genes was cloned in pKIapr as previously described (Ueki et al., 2017).

**FIGURE 1:**
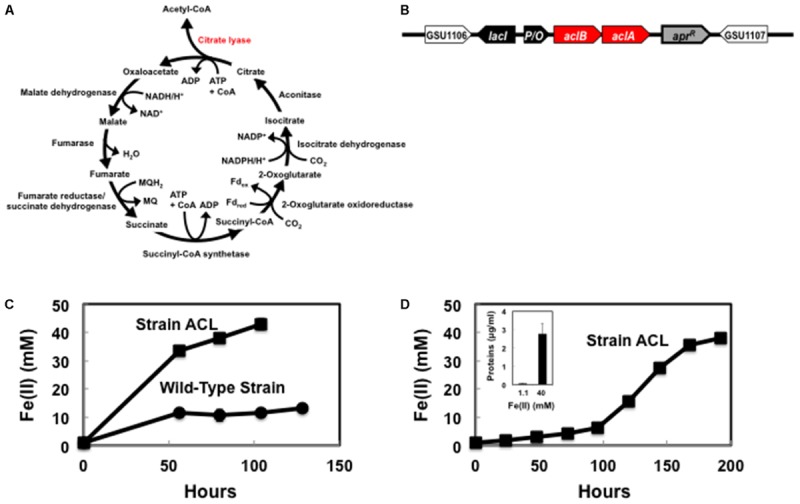
*Geobacter sulfurreducens* strain ACL capable of autotrophic growth. **(A)** Enzymes for reverse TCA cycle encoded in the native *G. sulfurreducens* genome and the requirement for ATP-dependent citrate lyase (in red) to complete the pathway. **(B)** Location of ATP-dependent citrate lyase genes within the *G. sulfurreducens* strain ACL chromosome. Citrate lyase genes: *aclA* and *aclB*; Lac repressor gene; *lacI*; *tac-lac* promoter/*lac* operator (IPTG-inducible): P/O; Apramycin resistance gene: *apr^R^*. **(C)** Fe(II) production from the reduction of Fe(III) with H_2_ as the electron donor following the first transfer of a 1% inoculum from cultures grown with acetate as the electron donor and Fe(III) as the electron acceptor. **(D)** Fe(II) production from the reduction of Fe(III) with H_2_ as the electron donor after a second transfer with a 2% inoculum of strain ACL from **(C)** into fresh medium. Inset-increase in protein concentration of strain ACL associated with Fe(III) reduction. The results are the means of duplicate cultures.

*Escherichia coli* NEB 10-beta (New England Biolabs) was used for plasmid preparation and grown in LB medium supplemented with appropriate antibiotics, as necessary. The synthetic ATP-citrate lyase genes were introduced into the chromosome adjacent to the gene (GSU1106) for citrate synthase in *G. sulfurreducens* strain DL-1 (**Figure 1**) with previously described methods (Ueki et al., 2017). Expression of the introduced genes was induced by isopropyl β-D-1-thiogalactopyranoside (IPTG) at a concentration of 1 mM.

Cells were grown with H_2_ as the electron donor and Fe(III) citrate as the electron acceptor as previously described (Coppi et al., 2004). The addition of acetate (1 mM) as a carbon source was necessary to maintain strain DL-1. Fe(II) production was analyzed with ferrozine (Lovley and Phillips, 1987). To estimate cell biomass, total cell extracts were prepared with B-PER Complete Bacterial Protein Extraction Reagent (Thermo Fisher Scientific) and the amount of protein was measured with the Bradford Protein Assay (Bio-Rad) as instructed by the manufacturer.

### Growth on Cathodes and Anodes

Cells were grown under anaerobic conditions in two-chambered bioelectrochemical devices with graphite electrodes and potentiostat control of the anode or cathode potential, as previously described with graphite electrodes of 65 cm^2^ (Nevin et al., 2009; Strycharz et al., 2011). Cathodes were poised at – 500 mV versus Ag/AgCl with fumarate (40 mM) as the electron acceptor. Anodes were poised at +300 mV versus Ag/AgCl with acetate (10 mM) as the electron donor.

### Confocal Microscopy

Biofilms on graphite cathodes were prepared using the LIVE/DEAD BacLight viability kit with antifade as previously described, except that isotonic wash buffer was used for rinsing and staining/destaining for 15 min (Nevin et al., 2008; Yi et al., 2009). Confocal scanning laser microscopy was imaged using a Leica TCS SP5 microscope, HCX PL FLUOTAR L 40× (numerical aperture 0.6) objective, and Leica LAS AF software (Yi et al., 2009).

## Results and Discussion

In order to construct a strain of *G. sulfurreducens* that did not require an organic carbon source, we built on previous analysis (Mahadevan et al., 2006; Feist et al., 2014) that suggested that *G. sulfurreducens* lacks a complete pathway for carbon dioxide fixation. Introduction of an ATP-dependent citrate lyase provides the one enzyme that *G. sulfurreducens* requires for a complete complement of enzymes necessary for a reverse TCA cycle (**Figure 1A** and Supplementary Table S1) that has the potential to make necessary biosynthetic precursors from carbon dioxide. Therefore, codon-optimized genes for the two subunits of this enzyme from *Chlorobium limicola* (Kanao et al., 2001) were introduced into the chromosome adjacent to the gene (GSU1106) for citrate synthase (**Figure 1B**). This strain was designated *G. sulfurreducens* strain ACL (ATP-dependent citrate lyase).

As previously reported (Coppi et al., 2004), the wild-type strain of *G. sulfurreducens* could not sustain metabolism with H_2_ as the electron donor and Fe(III) citrate as the electron acceptor in the absence of a carbon source (**Figure 1C**). However, strain ACL grew in repeated transfers into Fe(III) citrate medium with H_2_ provided as the electron donor and carbon dioxide as the sole carbon source (**Figure 1D**). Fe(III) reduction was accompanied with an increase in cell protein (**Figure 1D**, inset). These results demonstrated that expression of the citrate lyase was sufficient to confer the capacity for autotrophic growth.

**FIGURE 2:**
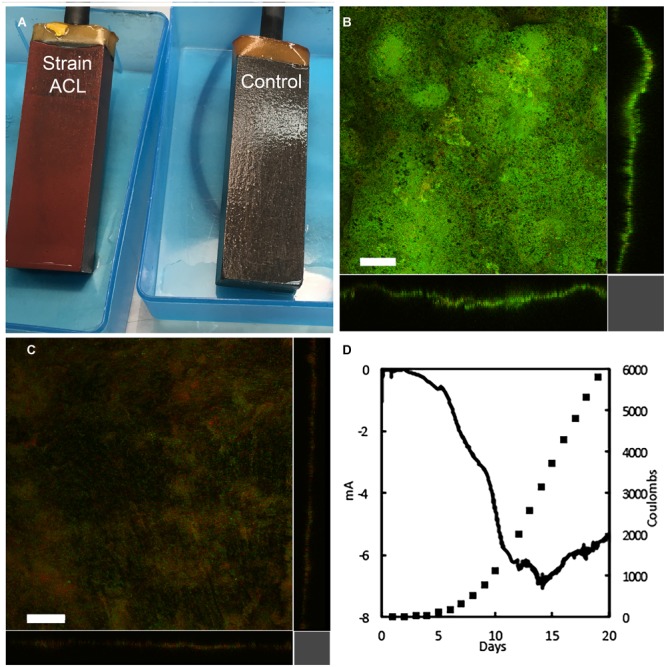
Cathode biofilms and current consumption. **(A)** Appearance of cathode biofilms of strain ACL and the control strain without citrate lyase genes. Confocal scanning laser micrographs of cathode biofilms of ACL **(B)** and wild-type **(C)** strains treated with Live/Dead stain. **(D)** Representative current consumption of ACL strain as current consumption rate (mA) and total cumulative coulombs consumed. Size bar = 50 μm.

Strain ACL grew well on cathodes in a medium in which cathodes were the sole electron donor, fumarate was the electron acceptor, and acetate was not added as a carbon source (**Figure 2**). The improved growth of strain ACL on the cathodes was visible as thick, red biofilms (**Figure 2A**). In contrast, wild-type cells grew poorly, even with the addition of an acetate carbon source (**Figure 2A**). Confocal scanning laser microscopy images of the cathodes colonized by strain ACL revealed thick biofilms (ca. 35 μm) that predominantly stained green with Live/Dead stain, suggesting that cells were metabolically active and had intact membranes (**Figure 2B**). Wild-type cathode biofilms were thinner (<10 μm), very patchy, and stained predominately red, suggesting that many of the cells were moribund (**Figure 2C**). The growth of strain ACL on cathodes was accompanied by current consumption that consistently maximized at more than 5 mA (**Figure 2D** and Supplementary Figure S2). This is more than 10-fold the maximum current consumption of ≤0.5 mA typically observed with the wild-type strain (Gregory et al., 2004; Strycharz et al., 2011).

The stoichiometry of total current consumption and the simultaneous recovery of electrons in the reduction of fumarate to succinate by mature cathode biofilms of strain ACL was determined over 6-h intervals in three separate bioelectrochemical devices. Coulombs of current consumed from the electrode and coulomb equivalents recovered in succinate production for the three replicate systems were: 40 coulombs consumed/41 equivalents recovered in succinate (recovery 102.5%); 103 coulombs consumed/96 equivalents recovered in succinate (recovery 93.2%); and 139 coulombs consumed/145 equivalents recovered in succinate (recovery 104.3%); yielding a mean and standard deviation of 100 ±6% for electron recovery in the triplicate studies.

Strain ACL generated currents comparable to the wild-type strain with acetate as the electron donor (Supplementary Figure S3). Carbon dioxide fixation by the reverse TCA cycle is not required during growth on acetate. Thus, possession of ATP-dependent citrate lyase would not be expected to enhance current production by strain ACL.

## Implications

*G. sulfurreducens* strain ACL is, to our knowledge, the first pure culture shown to grow anaerobically to high cell densities with electrons derived from a cathode as the sole electron donor. An additional benefit is that methods for genetic manipulation of *G. sulfurreducens* (Coppi et al., 2001; Chan et al., 2015, 2017; Ueki et al., 2016); a genome-scale understanding of many aspects of central metabolism (Mahadevan et al., 2006, 2011); and a developing model for electron transfer to extracellular electron acceptors (Lovley et al., 2011; Shi et al., 2016) are all available as tools to facilitate elucidation of the mechanisms of electron transfer from extracellular electron donors into cells.

In addition to expressing the required electron carriers, cells accepting electrons from electrodes must also balance carbon and electron flow in a manner that effectively supports growth. The complex regulatory networks in *G. sulfurreducens* evolved to recognize the availability of natural electron donors (Lovley et al., 2011). They may not be optimized to balance metabolism in wild-type cells presented with both acetate and cathodes, an unnatural source, as electron donors. The ability of strain ACL to grow autotrophically removes the complication of acetate as a second potential electron donor.

Genetic and metabolic modeling tools will aid in further evaluating this hypothesis of metabolic imbalance in wild-type cells and may lead to the design and construction of strains with enhanced ability for electron uptake on cathodes. For example, strains of *G. sulfurreducens* with faster rates of electron transfer to extracellular electron acceptors have been developed from the understanding of mechanisms for long-range electron transfer to extracellular electron acceptors and central metabolism (Izallalen et al., 2008; Tremblay et al., 2011; Malvankar et al., 2012a; Leang et al., 2013).

As the availability of renewable sources of electricity rises, and costs decline, feeding microorganisms electrons with an electrode is becoming an increasingly attractive possibility for the production of biofuels and other organic commodities (Lovley, 2011; Lovley and Nevin, 2013; Rosenbaum and Henrich, 2014; Tremblay and Zhang, 2015; May et al., 2016; Tremblay et al., 2017). Strain ACL may be the ideal chassis for such endeavors.

## Author Contributions

DL, KN, TU, MA, and DH conceived the study. TU constructed the ACL strain and evaluated autotrophic growth on hydrogen. KN and TW conducted the bioelectrochemical growth studies. DL, KN, and TU wrote the manuscript with modifications from all authors.

## Conflict of Interest Statement

The authors declare that the research was conducted in the absence of any commercial or financial relationships that could be construed as a potential conflict of interest.
