# Supplementary material for: Construction of a Geobacter Strain With Exceptional Growth on Cathodes
Source: Front Microbiol. 2018 Jul 13;9:1512. doi: 10.3389/fmicb.2018.01512 (PMC6053493; doi:10.3389/fmicb.2018.01512)
Supplement: Supplementary file 1 [file Data_Sheet_1.DOCX]

**Supplementary Information**

**Construction of a *Geobacter* Strain with Exceptional Growth on Cathodes**

Toshiyuki Ueki^1^, Kelly P. Nevin^1^, Trevor L. Woodard^1^, Muktak A. Aklujkar^1,2^, D.E. Holmes^1,3^, and Derek R. Lovley^1^

^1^Department of Microbiology, University of Massachusetts, Amherst, MA 01003

^2^Present address: Applied Biological Materials Inc., Richmond, BC., Canada

^3^Department of Physical and Biological Sciences, Western New England University, Springfield, MA

>aclBA

GGATCCAAGGAGAGCACGTAAC

**ATGGCCAAGATCCTCGAAGGCCCCGCCATGAAGCTGTTCAACAAGTGGGGCATCCCGGTTCCCAACTACGTCGTTATCATGGACCCGAACCGGCTGGAGCAGCTCGGCGAGGCCAACAAGTGGCTGCGCGAGTCCAAGCTCGTGGTCAAGGCCCATGAGGCCATCGGCGGCCGGTTCAAGCTCGGCCTGGTCAAGCTCGGCCTGAACCTCGAAGAGGCCGTGGCCGCCTCCCGGGAGATGATCGGCCGGGAGATCGGCACCGCCGTCATCCGCCAGGTGATCGTGGCCGAGATGCTGGACCACGACGAGGAGTACTACCTCTGCATCAACGGCAACCGGGACGGCGCCGAGGTCCTCCTGTCCAACCGCGGCGGCGTGGACATCGAGGAGAACTGGGACACCGTCCGGCGCCTGTTCATCCCCATCGACGAGAACCCCTCCGTGGAGCGCCTCACCGAGTTCGCCGCCGAGGCCGGCTTCACCGGCGAGATCGCCGAGCGGGTGGGCAAGATCGTCTCCCGCCTGGTGCTCTGCTTCGACAACGAGGACGCCCAGTCCATCGAGATCAACCCCCTGGTCATCCGGAAGTCCGACATGCGCTTCGCCGCCCTCGACGCCGTGATGAACGTGGACTACGACGCCCGGTTCCGCCATGCCGACTGGGACTTCAAGCCCGTGTCCGAGATCGGCCGTCCCTTCACCGAGGCCGAGCTGCAGATCATGGAGATCGACGCCCGCATCAAGGGCTCCGTCAAGTTCGTGGAGGTCCCCGGCGGCGAGATCGCCCTCCTGACCGCCGGTGGCGGCGCCTCCGTGTTCTACTCCGACGCCGTGGTGGCCCGTGGCGGCACCATCGCCAACTACGCCGAGTACTCCGGCGACCCCTCCGACTGGGCCGTGGAGGCCCTGACCGAGACCGTGTGCGCCCTCCCCAACATCAAGCACATCATCGTCGGCGGCGCCATCGCCAACTTCACCGACGTGAAGGCCACCTTCAACGGCATCATCAACGGCTTCCGGGAGTCCAAGTCCAAGGGCTACCTGGAGAACGTCAAGATCTGGGTGCGTCGGGGCGGCCCCAACGAGAACCAGGGCCTGGCCGCCATCCGCAAGCTCCAGGACGAGGGCTTCGACATCCATGTCTACGACCGCTCCATGCCCATGACCGATATCGTGGATCTGGCCCTCAACTCGTAA**

GGCATCAGGAGAGAAAGAATC

**ATGTCCATCCTTGCCAACCGCGACACCCAGGTGGTTATCATCGGCGGCGTTGCCGGCCTTAACGCCGCCCGCCGCATGGCCCAGTTCGACTTCCTCATCAACCGGCCCCTGTCCGTGCAGGCCTTCGTCTACCCGCCCGAGGAAGGCCAGCAGAAGGAGATCTACCGGGGCGGCGAGCTGAAGAACGTGCCCGTCTACTCCTCCCTGGAGGACGCCCTGGCCGAGAACCCCGGCATCAACACCGCCCTGATCTACATCGGCGCCAACCGCGCCTACCAGGCCGCCAAAGAGGCCCTGGAGGCCTCCTCCATCAAGCTGGTGTCCATGATCACCGAGGGCGTCCCGGAGAAGGACGCCAAGCGCCTCCGCAAGCTGGCCATCGAGGCCGGCAAGCTCTTCAACGGCCCCTCCTCCATCGGCATCATGTCCGCCGGCGAGTGCCGGCTCGGCGTGATCGGCGGCGAGTTCAAGAACCTCAAGCTGTGCAACCTGTACCGCTCCGGCTCCTTCGGCGTCATCACCAAGTCCGGCGGCCTCTCCAACGAGGCCATGTGGCTGTGCGCCCAGAACGGCAACGGCATCACCACCGCCGTGGCCATCGGCGGCGACGCCTACCCCGGCACCGACTTCGTCACCTACCTGGAGATGTTCGAGAAGGACCCCGAGACCAAGGCCGTGGTCATCGTGGGCGAGGTCGGCGGCACCCTGGAGGACGAGGCCGCCGAGTGGCTGGCCGCCGAGAAGCGGCGCATCCGGCTGGTGGCCACCATCGGCGGCACCTGCCAGGAAGTGCTGCCGCAGGGCATGAAGTTCGGCCATGCCGGCGCCAAGGAAGGCAAGAAGGGCGTGGGCTCCGCCCGTGCCAAGATGAACGCCCTCCGGGACGCCGGCGCCCTGGTGCCCGACACCTTCGGTGGCCTCTCCAAGTGCATCAAGCAGGTCTACGAGGAGCTGCTCGCCGACGGCTCCATCAAGCCGGAGCCGGAGATCGACGAGGCCCTCCTGCCGGAGCTGCCCCTGAAGGTCCAGGAGATCATGAAGCAGGGCGAGGTGATCGTCGAGCCCCTCATCCGCACCACCATCTCCGACGACCGGGGCGAGGAGCCCCGCTACGTGGGCTACGCCGCCTCCGAGCTGTGCGAGAAGGGCTACGGCATCGAGGACGTCCTCTCCCTCCTGTGGTCCAAGAAGCTGCCCTCCCGGGAGGAGTCCGAGATCATCAAGCGCATCATCATGATCTCCGCCGACCATGGCCCGGCCGTGTCCGGCGCCTTCGGCGCCATCATCGGCGCCTGCGCCGGCATCGACCTCCCCCAGGCCGTGTCCGCCGGCATGACCATGATCGGCCCCCGCTTCGGTGGCGCCGTGACCAACGCCGGCAAGTACTTCAAGTACGGCGTCAAGGAGTTCCCCAACGACATCCCCGGCTTCCTGTCCTGGATGAAGCAGAACGTGGGCCCGGTCCCGGGCATCGGCCACCGGGTGAAGTCCGTCAAGAACCCCGACAAGCGCGTGAAGTACCTCGTGGACTACGTCAAGAACCACACCTCCCTGCACACCCCGTGCCTCTCCTACGCCCTGGAGGTCGAGAAGATCACCACCTCCAAGAAGGACAACCTCATCCTGAACGTGGACGGCACCATCGGCTGCATCCTCGTGGACCTCGACTTCCCCGAGCAGTCCCTCAACGGCTTCTTCGTGCTGGCCCGGACCATCGGCATGATCGGCCACTGGATCGACCAGACCACCCAGGGCTCCAAGCTCATCCGCCTGTACGACTACCTCATCAACTACGCCGTCAAGGAAGAACGCGAAGTGCCCGTGAAGAAGTAA**

GAATTC

Figure S1. Sequence of *aclBA* genes with codon optimization for *G. sulfurreducens*. Coding regions are indicated in bold. Ribosome binding sites are underlined. GGATCC (BamHI) and GAATTC (EcoRI) at the 5’ end and 3’ end, respectively, were used for cloning.

**Supplementary Table 1. Enzymes and their gene numbers in the reverse TCA cycle.**

| Enzyme | Category | Gene number |
| --- | --- | --- |
| Citrate lyase | EC:2.3.3.8 | Clim_1231-1232 |
| Aconitase | EC:4.2.1.3 | GSU0846, GSU1660, GSU2445 |
| Isocitrate dehydrogenase | EC:1.1.1.42 | GSU1465 |
| 2-Oxoglutarate oxidoreductase | EC:1.2.7.3 | GSU1467-70 |
| Succinyl-CoA synthetase | EC:6.2.1.5 | GSU1058-9 |
| Fumarate reductase/  succinate dehydrogenase | EC:1.3.5.1 | GSU1176-8 |
| Fumarase | EC:4.2.1.2 | GSU0994 |
| Malate dehydrogenase | EC:1.1.1.37 | GSU1466 |

**
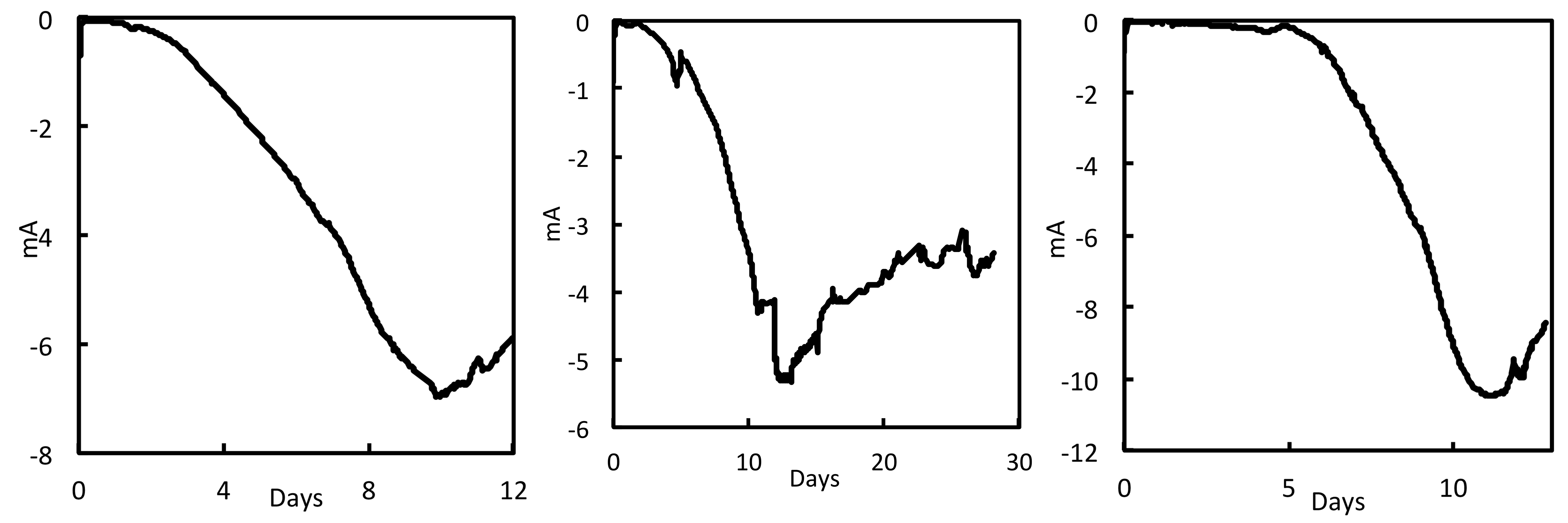
**

Supplementary Figure S2. Additional time courses of strain ACL current consumption.

Supplemental Figure 3. Representative current produced by strain ACL and the control wild-type strain over time when acetate was provided as the electron donor. Cells were grown under anaerobic conditions in two-chambered systems with a graphite anode poised at +300 mV versus Ag/AgCl as previously described (Nevin et al., 2009).

**References**

Nevin, K.P., Kim, B.-C., Glaven, R.H., Johnson, J.P., Woodard, T.L., Methé, B.A., Didonato Jr, R.J., Covalla, S.F., Franks, A.E., Liu, A., and Lovley, D.R. (2009). Anode biofilm transcriptomics reveals outer surface components essential for high current power production in Geobacter sulfurreducens fuel cells. *PLoS ONE* 4**,** e5628.
